# Primary Neuroendocrine Tumor of the Parotid Gland: A Case Report and a Comprehensive Review of a Rare Entity

**DOI:** 10.1155/2016/6971491

**Published:** 2016-08-16

**Authors:** Olga Martínez-Sáez, Javier Molina-Cerrillo, Carmen Moreno García del Real, Rafael Barberá Durban, Juan J. Díez, Teresa Alonso-Gordoa, Enrique Grande Pulido

**Affiliations:** ^1^Department of Medical Oncology, Ramón y Cajal Hospital, University of Alcalá de Henares, Carretera Colmenar Viejo, Km 9.1, 28034 Madrid, Spain; ^2^Department of Pathology, Ramón y Cajal Hospital, University of Alcalá de Henares, Carretera Colmenar Viejo, Km 9.1, 28034 Madrid, Spain; ^3^Department of Otorhinolaryngology, Ramón y Cajal Hospital, University of Alcalá de Henares, Carretera Colmenar Viejo, Km 9.1, 28034 Madrid, Spain; ^4^Department of Endocrinology, Ramón y Cajal Hospital, University of Alcalá de Henares, Carretera Colmenar Viejo, Km 9.1, 28034 Madrid, Spain

## Abstract

Neuroendocrine tumors (NETs) comprise a heterogeneous group of malignancies from cells derived from the neural crest with neuroendocrine differentiation. Despite the differences in the site of origin, nomenclature, biological behavior, and functional status, NETs share certain ultrastructural and immunohistochemical features. NETs are relative rare tumors with an annual incidence of 5.76 new cases per 100.000 inhabitants and they usually appear in the gastrointestinal tract or in the pulmonary system. Head and neck NETs are uncommon with limited information regarding frequency, most of them showing small cell carcinoma features. NETs that arise from the salivary glands are exceedingly rare. Regardless of their low frequency, it is imperative to accurately differentiate these tumors from the much more common squamous cell carcinomas and from metastasis from another primary tumor due to the completely different therapeutic approaches and prognosis. The diagnosis is based on the recognition of the typical neuroendocrine architecture and immunohistochemical staining and on an exhaustive work-up. Hereby, we report a case of a moderately differentiated NET of the parotid gland that was treated with a complete parotidectomy. We summarize the clues that led to the final diagnosis and major strategies that were employed to manage the patient. We also perform a comprehensive review of the scarce available literature on this topic.

## 1. Introduction

As an exceedingly rare entity, NETs of the head and neck region represent a diagnostic and therapeutic challenge in the routine practice. A complete work-up is necessary to rule out a metastatic origin of the tumor, since NETs are much more common in other parts of the body [1, 2]. An adequate subclassification of NETs in the head and neck area regarding the degree of differentiation is required to predict the clinical behavior and to support the treatment decision-making. Clinical-morphological correlations in large series of cases are necessary to provide clear diagnostic categories and to define the best therapeutic options [3].

## 2. Case Report

A 67-year-old woman was referred to our institution's maxillofacial surgery department with a 3-month history of asymptomatic growing mass in the left parotid area. The patient's medical history included hypertension, dyslipidaemia, and chronic bronchitis. Physical examination revealed a nodule of 1.5 cm of diameter in the parotid gland. There were no cervical palpable lymphadenopathies and no intraoral lesions, and the facial nerve was preserved. A fine-needle aspiration biopsy was subsequently performed. The cellular extensions showed abundant cellularity with basaloid appearance with scant cytoplasm. Neither necrosis nor mitosis was observed at the tumor sample.

**Figure 1:**
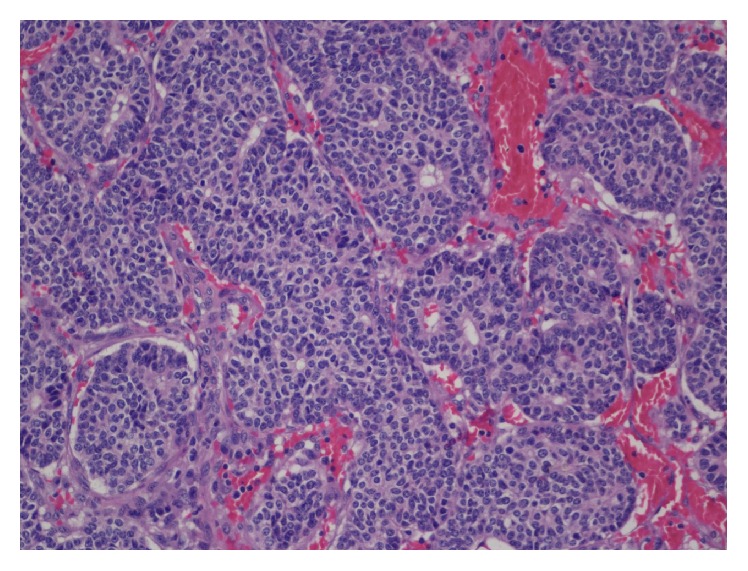
Light microscopy shows monomorphous round cells with salt-and-pepper chromatin arranged mostly in nests with a cribriform pattern that formed rosette-like structures (hematoxylin and eosin stain, original magnification ×20).

**Figure 2:**
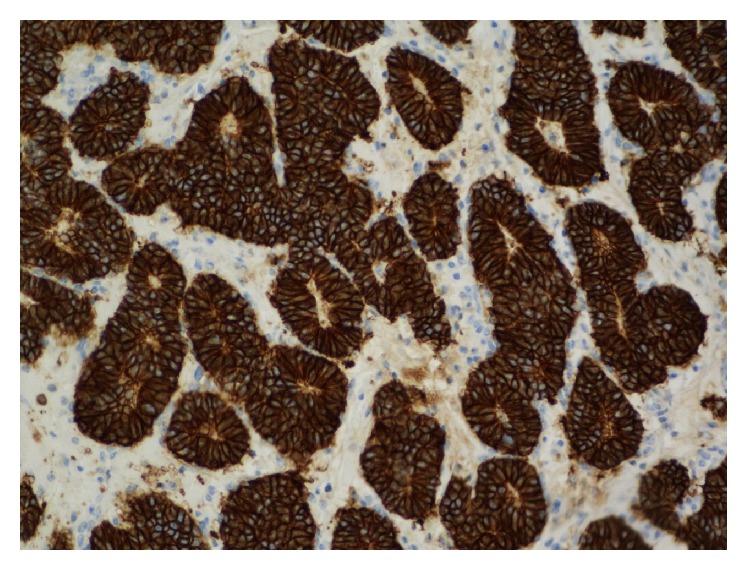
Further immunohistochemical analysis shows strong positive staining for CD56 (original magnification ×20).

A complete parotidectomy was then performed. The macroscopic examination showed a well-circumscribed elastic white mass located in the superficial parotid lobe that measured 1.6 cm in its greatest dimension. Under the light microscopy it consisted of an epithelial infiltrating neoplasm with an organoid pattern of growth. It showed monomorphous round cells with salt-and-pepper chromatin arranged mostly in nests with a solid or cribriform pattern that formed frequent rosette-like structures (Figure 1). Vascular embolization and perineural infiltration were observed. The immunohistochemical study supported the neuroendocrine origin with positivity for CD56 (Figure 2). CK AE1/AE3 was also positive. Staining was negative for CK 5/6, CK7, CK20, calponin, synaptophysin, and chromogranin. The mitotic index was around 10%. These features were compatible with atypical carcinoid according to the World Health Organization (WHO) classification of head and neck NETs and with a well differentiated, grade 2, NET, according to the European Neuroendocrine Tumour Society (ENETS) and WHO classification of gastroenteropancreatic NETs. The pathological stage was pT1Nx according to TNM/AJCC classification.

A clinical and radiographic work-up was performed after surgery. Computed tomography (CT) of the neck, chest, abdomen, and pelvis, positron-emission tomography (PET)/CT, and octreoscan were all negative. The absence of any other tumor confirmed the diagnosis of a primary neuroendocrine tumor of the salivary gland.

No further treatment was offered to the patient after surgery. No recurrent disease has been observed after 7 months of following up.

## 3. Discussion

Primary NETs of the head and neck are exceedingly rare and there is a considerable debate regarding the best practical approach for their management. The current WHO classification for lung NETs recognizes four major types based on mitotic rate and extent of necrosis: carcinoid, atypical carcinoid, small cell carcinoma, and large cell neuroendocrine carcinoma [3, 4]. However, some clinicians have argued that the term* atypical carcinoid* implies a close relationship with the more indolent* typical carcinoid*, while atypical carcinoids are more aggressive and highly metastatic malignancies. Meacham et al. proposed the terms* well differentiated*,* moderately differentiated*, and* poorly differentiated* to best classify these tumors [2].

Recently, the WHO recommended a new classification system for gastrointestinal NETs regardless of the primary tumor origin. This classification splits tumors in grades based on tumor proliferation: well differentiated NETs and poorly differentiated neuroendocrine carcinomas (NECs). Well differentiated NETs were further separated into 2 subgroups: grade 1, which are tumors having a proliferative index of <2% (or mitotic counts of ≤2 per 10 high power fields) and are equivalent to carcinoid tumor, and grade 2, with proliferative indices ranging from 2 to 20% (or mitotic counts of 3–20 per 10 high power fields); the grade 3 NEC has proliferative indices of >20% (or mitotic counts >20 per 10 high power fields) and was subclassified as large cell or small cell types. We are going to use this last classification in our paper, although its proper application to head and neck NETs has not been determined yet [1].

Primary NETs of the head and neck are more frequently derived from the larynx but only account for 0.5–1% of all tumors at this location [5]. In the salivary glands, most reported NETs are small cell carcinomas (SmCC), constituting around 2% of all tumors, with some cases of large cell NECs and well differentiated, grade 1, NETs, but only few previously reported well (moderately) differentiated, grade 2, NETs [1, 2, 4, 6, 7].

The reported male-to-female ratio for well differentiated, grade 2, NETs of the larynx is 3-to-1, and most patients have been heavy smokers [6].

Poorly differentiated NECs seem to have a strong male predominance and correlation with a smoking habit. This association has not been seen in well differentiated tumors [8].

The pathological diagnosis of the NET in the head and neck area may be difficult just because of the low frequency of these tumors in that location. The diagnosis is based on histological, ultrastructural, and immunohistochemical criteria, which may be overlooked or misdiagnosed especially in small biopsy samples. For that reason, a large core needle biopsy, rather than a fine-needle aspiration, is preferred for the diagnosis. Due to its rarity, an adequate metastatic work-up is also imperative for the diagnosis of a primary tumor. The morphological characteristics of NETs are the organoid pattern of growth (nests, cords, trabeculae, glands, or rosette-like structures), the presence of neurosecretory cytoplasmatic granules and finely granular chromatin, and the absence of keratinization [4]. The differentiation between well and poorly differentiated tumors is essential to adequately predict the clinical behavior. Unlike NETs, NECs usually appear with a solid growth pattern, a less frequent gland formation, and a marked cellular pleomorphism, with abundant mitosis and necrosis. Vascular and perineural invasions are also common findings in poorly differentiated NECs [1, 4].

The immunodiagnostic is based on immunohistochemical proof of a simultaneous epithelial and neuroendocrine differentiation. The tumor cells stain positively with broad-spectrum cytokeratin and they often display the characteristic punctate paranuclear dot staining [9]. Some tumors will also react with cytokeratins 7 and 20 (CK-7 and CK-20 positivity). NETs are staining for at least one of the most known neuroendocrine markers [synaptophysin, chromogranin, and CD56 neural cell adhesion molecule (NCAM), CD57 (Leu-7), and neuron specific enolase (NSE)] [1, 4, 7].

Immunohistochemical staining is helpful to rule out the differential diagnosis of a primary NET at the parotid gland from a distant metastasis from a NET with another primary origin. For example, thyroid transcription factor-1 (TTF-1) is a sensitive marker for lung SmCC (positive in 80%–100%), so its absence in tumor cells is of value to exclude a metastatic NEC of the lung. TTF-1 also helps to rule out Merkel cell carcinoma, where it is consistently negative. CK20 positive staining observed in primary salivary carcinomas may help to exclude metastatic small cell carcinoma of pulmonary origin, which typically does not stain with CK20. On the other hand, CK20 negativity practically rules out Merkel cell carcinoma (Table 1) [3, 9, 10].

Immunohistochemical study is also useful to distinguish other malignant small round cell neoplasms that may be considered in the differential diagnosis, as olfactory neuroblastoma, sinonasal undifferentiated carcinoma, basaloid squamous carcinoma, non-Hodgkin lymphoma, and paraganglioma (Table 2) [1, 2, 9, 11, 12].

Besides the immunohistological analysis, the clinical exam and radiologic imaging with CT, PET/CT, and octreoscan are basic to rule out an alternative primary tumor [13]. Moreover, octreoscan may demonstrate the presence of somatostatin receptors in tumor cells that can be amenable to radiometabolic treatment [4, 14].

As in other primary origin NETs, prognosis will depend on the histological subtype and the stage at diagnosis. Well differentiated NETs of head and neck have been classically thought to be indolent; however, recent data have shown that their biologic behavior is significantly worse than other NETs in the body and locoregional metastasis reported rates are around 30% [2, 8, 14].* Woodruff and Senie* found that well differentiated, grade 2, NETs of the larynx had an aggressive nature with metastasis to neck nodes in 43% of cases and to distant sites in 44% and five-year survival of 48% [6]. Poorly differentiated NECs are highly malignant tumors [2]. Metastases were most often found in cervical nodes, liver, bone, skin, and lung. Survival rates were similar to those for pulmonary SmCC, with 2- and 5-year survival rates of only 16% and 5%, respectively [2, 15].

The therapeutic approaches to NETs of the head and neck area vary according to the histological type and disease stage [4]. The mainstay of treatment to well differentiated NET is surgical resection. There is a lack of agreement about the need of elective neck dissection in these cases. Some authors recommend further lymphadenectomy mostly in well differentiated, grade 2, NETs due to the high likelihood of nodal metastasis, while others do not [16]. In addition, some authors advocate that adjuvant radiation conferred no benefit in the treatment of (moderately) well differentiated NETs of the larynx despite the established role of radiotherapy in more frequent tumors of the head and neck area [6]. This fact emphasizes the need to distinguish these tumors from more radiation-sensitive squamous cell carcinomas. Chemotherapy seems to be ineffective as well, although the responsiveness of these lesions to the different chemotherapy regimens has not been well documented. By contrast, chemoradiotherapy is the treatment of choice of poorly differentiated NECs [6, 12]. The chemotherapeutic regimens usually included platinum in combination with other agents (mostly etoposide, but also 5-fluorouracil, ifosfamide, paclitaxel, methotrexate, gemcitabine, vincristine, vinblastine, or bleomycin). In our patient, no further treatment was offered after surgery due to the lack of evidence behind the realization of a neck dissection and the use of radiation or chemotherapy in the adjuvant context of well differentiated NETs. The extensive work-up did not show any evidence of disease, neither local or regional nor distant, which supported the decision of not adding any treatment.

Expression of somatostatin receptors through a high uptake of octreotide in scintigraphy could be useful to select tumors that could benefit from the systemic use of somatostatin analogues [17]. Due to the low incidence of these tumors in the head and neck location there is a lack of prospective evidence behind the use of everolimus, peptide receptor radionuclides therapy (PRRT), multikinase inhibitors, or novel chemotherapeutic agents like temozolomide and/or capecitabine.

In conclusion, the present case report emphasizes that the proper pathologic identification of primary NET in the head and neck area and their differentiation from squamous cell carcinoma or a metastatic tumor is necessary because prognosis and management of these patients are not the same. It is also imperative to correctly distinguish between NET subtypes, due to the fact that well and poorly differentiated NECs present different clinical behavior and require different treatment approaches. Because of the rarity of this entity, an appropriate registry of the cases is highly needed to gather experience in its management.

## Figures and Tables

**Table 1:** Immunohistochemical features that help in the differential diagnosis between NEC from the parotid, SmCC of the lung, and Merkel cell carcinoma.

	NEC parotid origin	SmCC lung	Merkel cell carcinoma
TTF-1	−	+	−
CK20	+/−	−	+
CK7	+/−	+	−

**Table 2:** Immunohistochemical staining of some malignant round cell tumors. SmCC: small cell carcinoma, NB: neuroblastoma, SYP: synaptophysin, CgA: chromogranin A, EMA: epithelial membrane antigen, CK: cytokeratin, TTF-1: thyroid transcription factor-1, NSE: neuron specific enolase.

	SmCC	Melanoma	NB	Sinonasal undifferentiated carcinoma	Lymphoma	Paraganglioma	Basaloid squamous carcinoma
EMA	+	−	−	+		−	+
CK	+	−	+/−	+		−	+
CD99	+/−	−	−	+	+/−		−
TTF-1	+/−	−	−	−		−	−
CD45	−	−	−	−	+	−	−
S100	−	+	−	+/−		+/−	−
HBM45	−	+	−	−	−	−	−
CD56	+	−	+	+/−		+	−
CD57	+	−	+	+/−		+	−
SYP	+	−	+	+/−			−
CgA	+	−	+	+/−		+	−
NSE	+	−	+	+/−		+	−
